# Supplementary material for: CNKSR1 serves as a scaffold to activate an EGFR phosphatase via exclusive interaction with RhoB-GTP
Source: Life Sci Alliance. 2021 Jun 29;4(9):e202101095. doi: 10.26508/lsa.202101095 (PMC8321701; doi:10.26508/lsa.202101095)

Full images of western blotting. The area enclosed in red squares are shown in main figures.

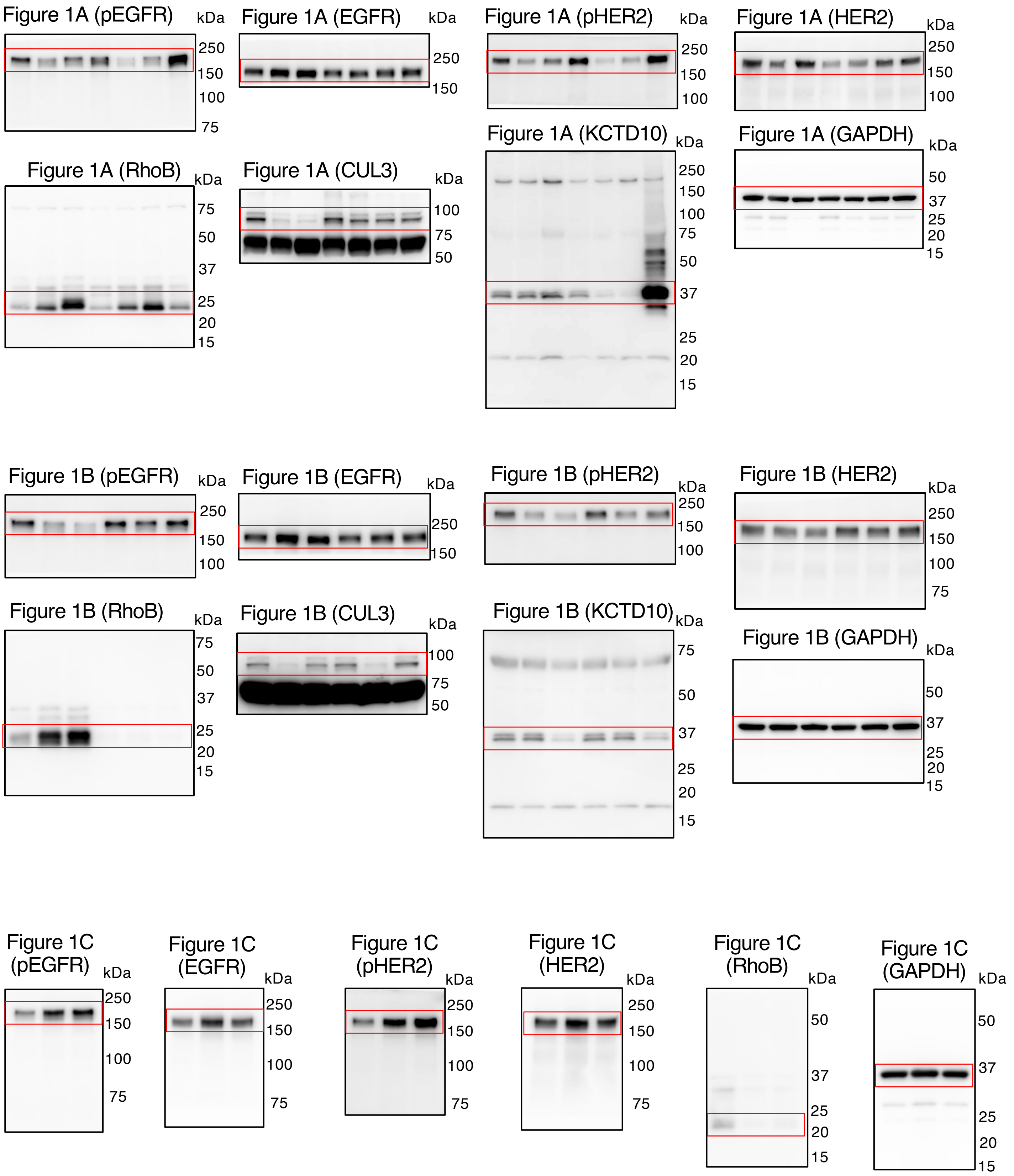

Nishiyama, Maekawa *et al.*

Full images of western blotting. The area enclosed in red squares are shown in main figures.

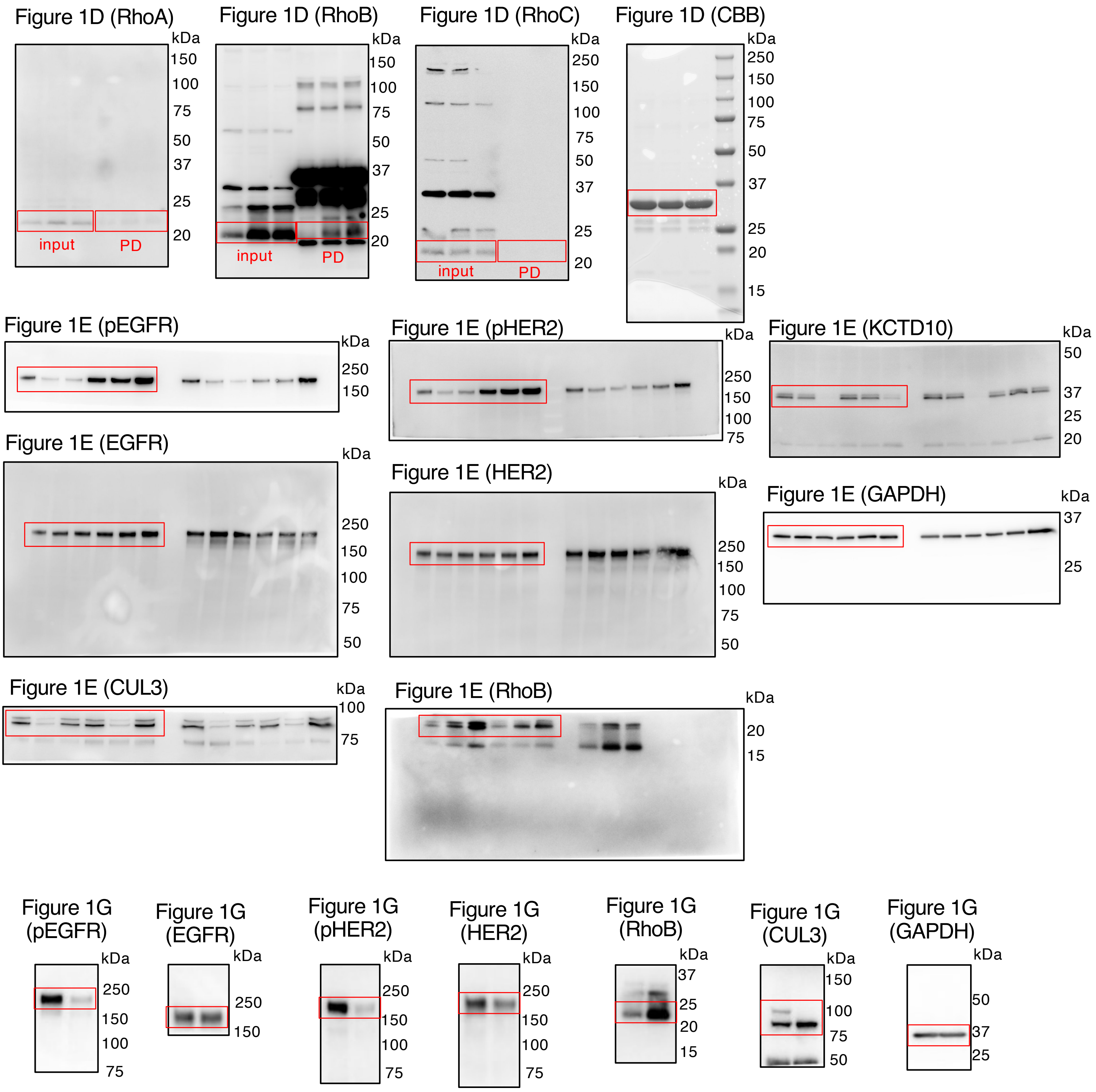

Full images of western blotting. The area enclosed in red squares are shown in main figures.

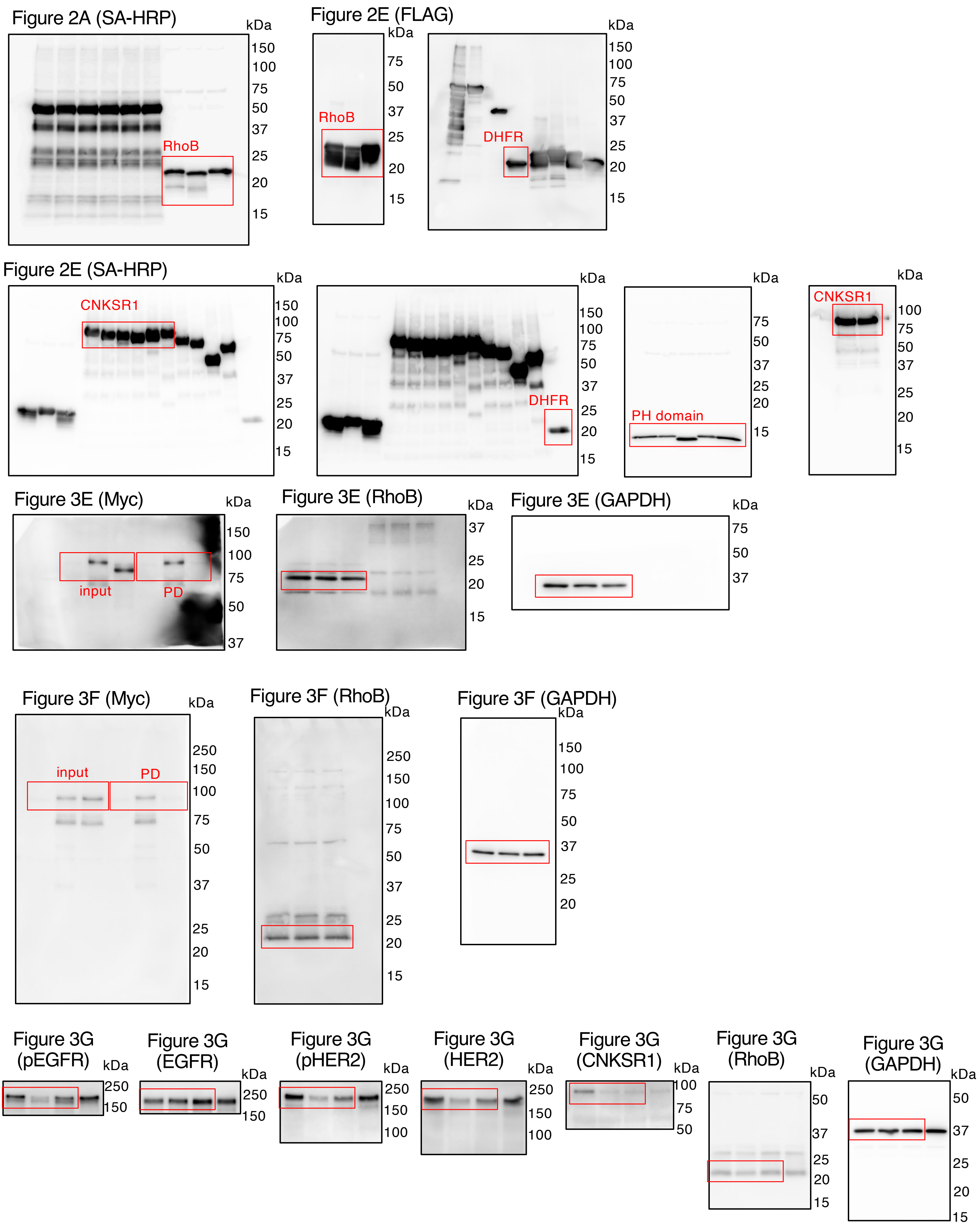

Nishiyama, Maekawa *et al.*

Full images of western blotting. The area enclosed in red squares are shown in main figures.

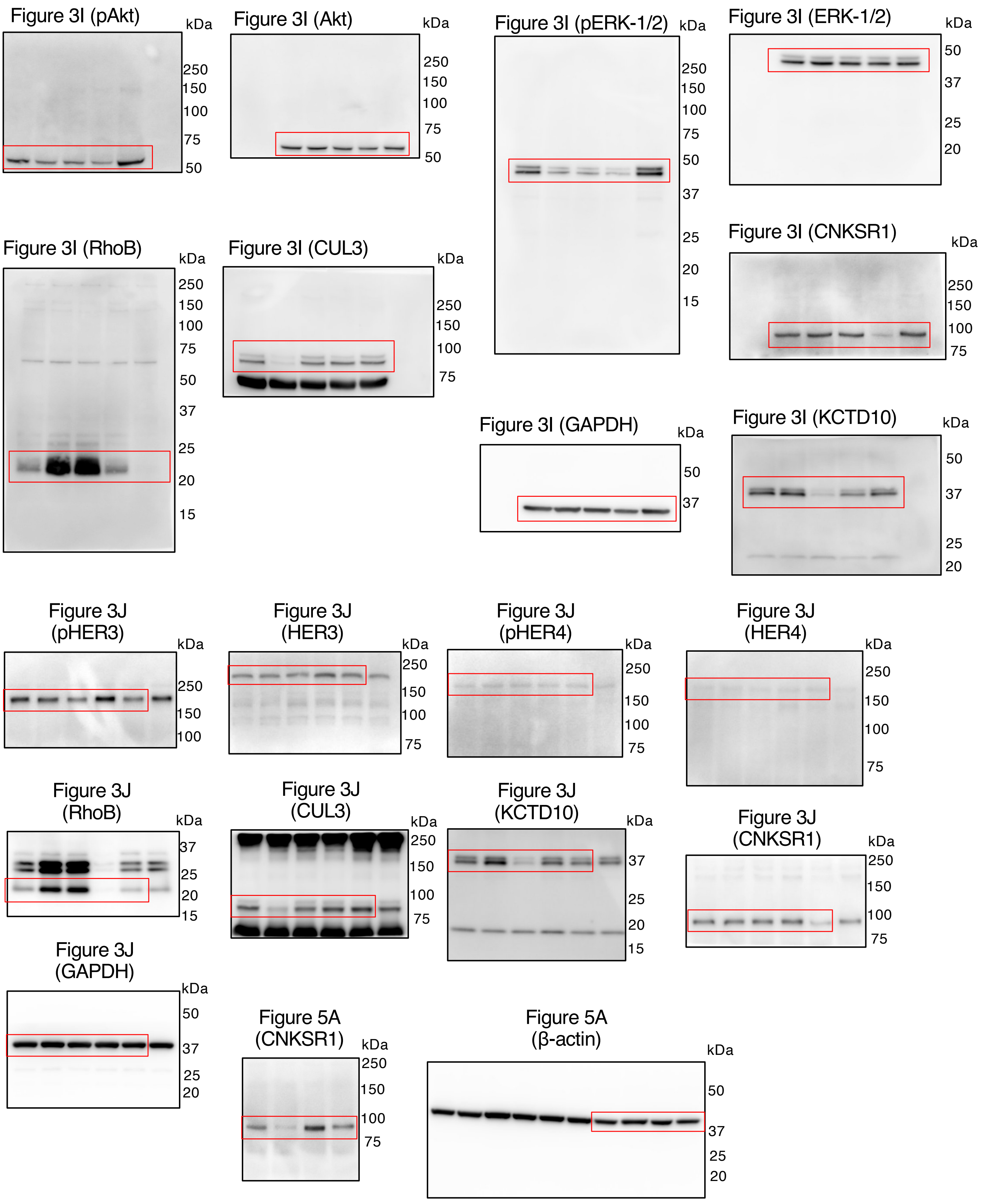

Nishiyama, Maekawa *et al.*

Full images of western blotting. The area enclosed in red squares are shown in main figures.

Figure 6A (pEGFR)

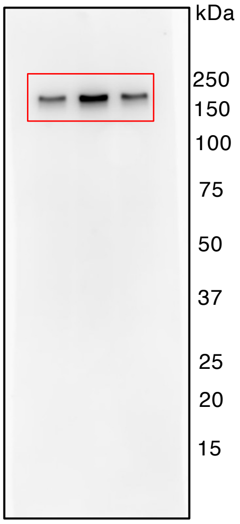

Figure 6A (EGFR)

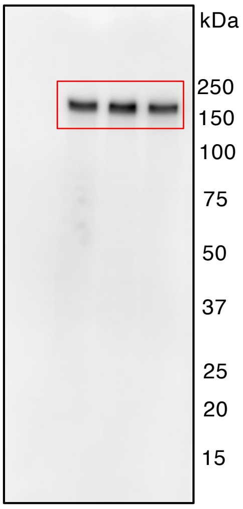

Figure 6A (pHER2)

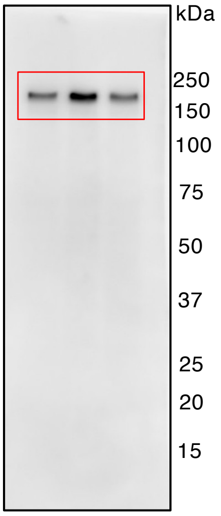

Figure 6A (HER2)

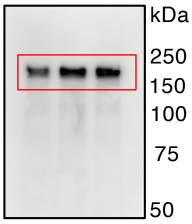

Figure 6A (Myc)

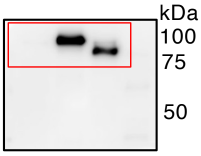

Figure 6A (RhoB)

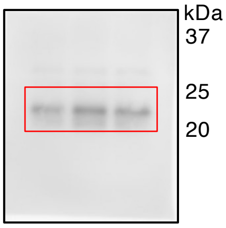

Figure 6A (GAPDH)

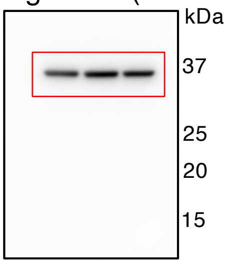

Figure 6B (pEGFR)

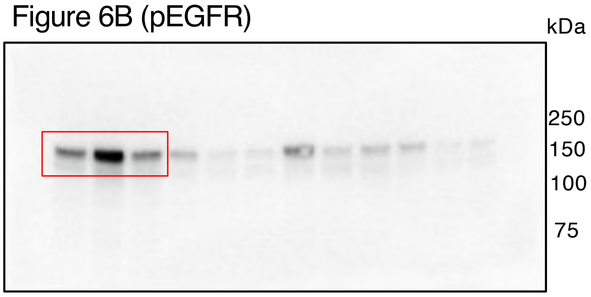

Figure 6B (pHER2)

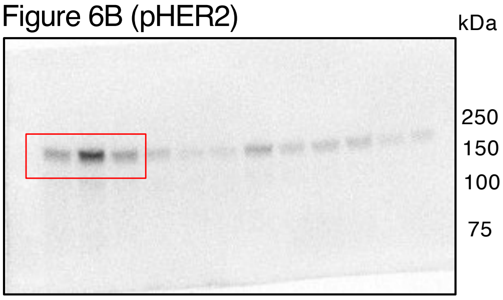

Figure 6B (EGFR)

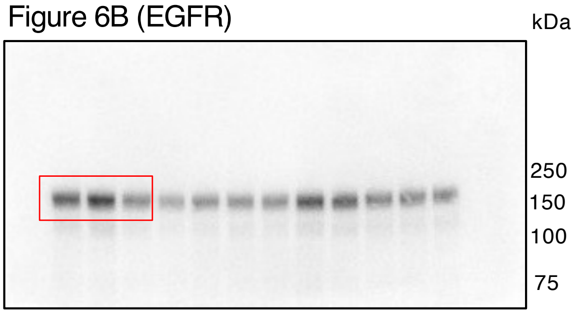

Figure 6B (HER2)

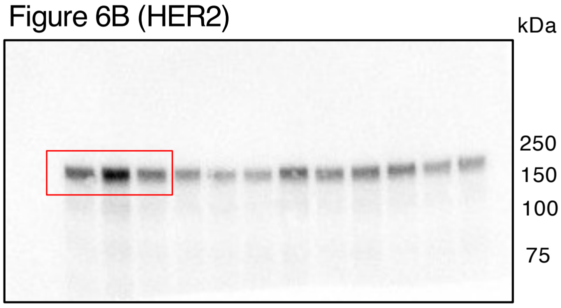

Figure 6B (CNKSR1)

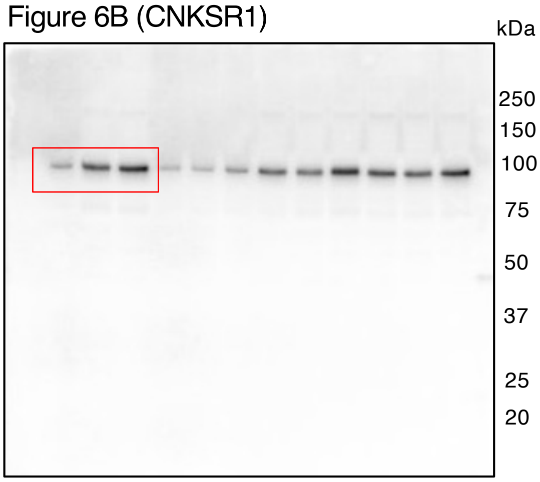

Figure 6B (RhoB)

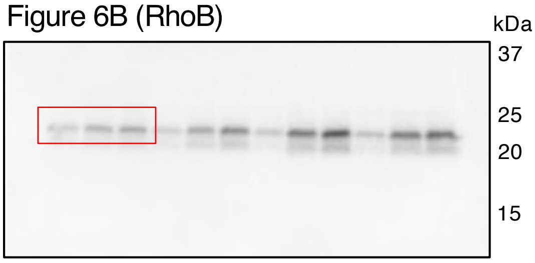

Figure 6B (GAPDH)

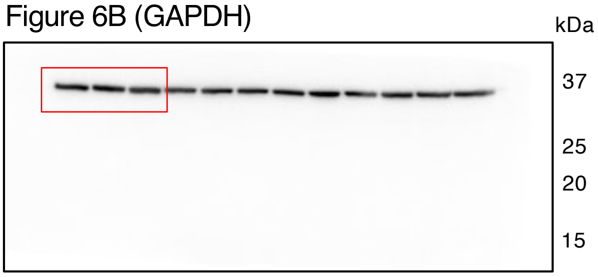

Nishiyama, Maekawa *et al.*

Full images of western blotting. The area enclosed in red squares are shown in main figures.

Figure 6E (pEGFR)

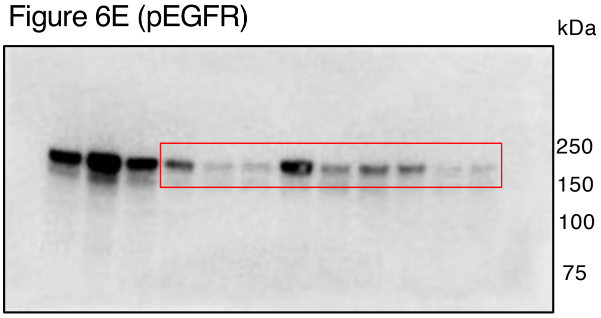

Figure 6E (EGFR)

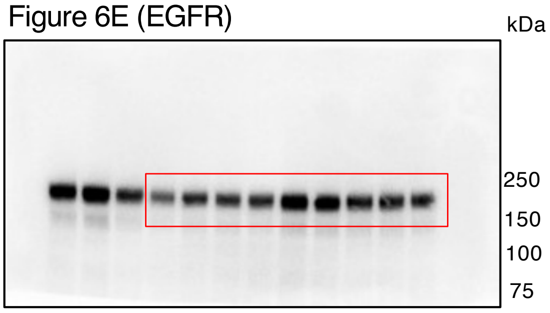

Figure 6E (pHER2)

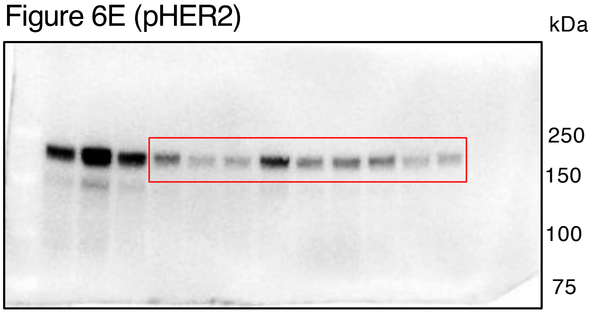

Figure 6E (HER2)

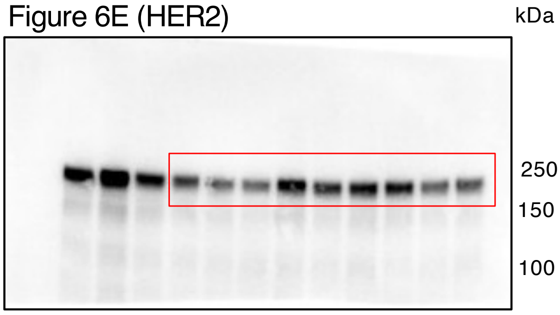

Figure 6E (GAPDH)

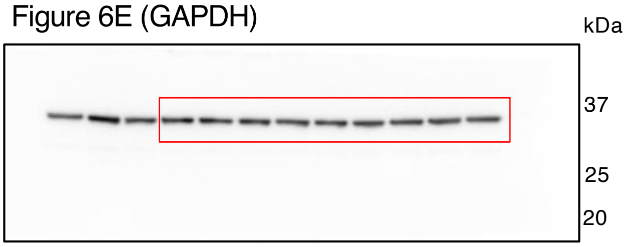

Figure 6E (RhoB)

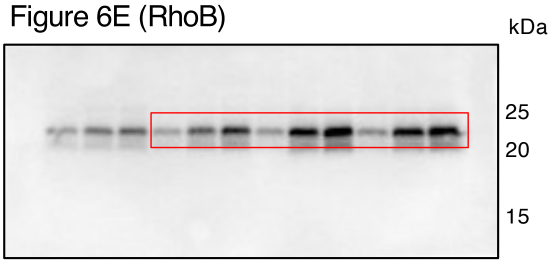

Figure 6E (CNKSR1)

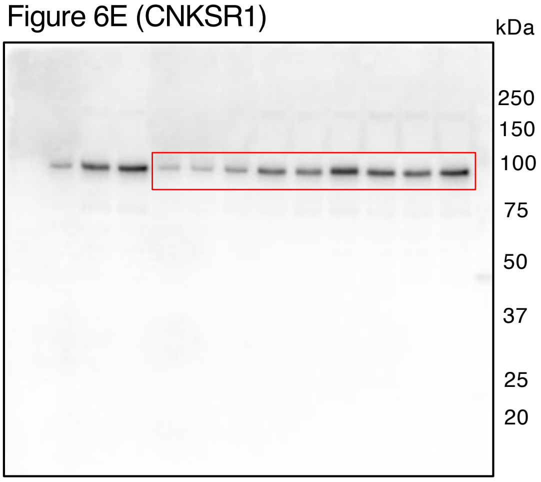

Figure 6E (CUL3)

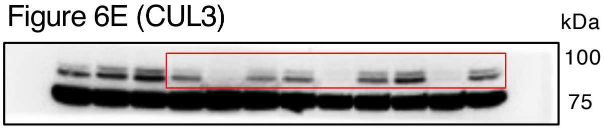

Figure 6E (KCTD10)

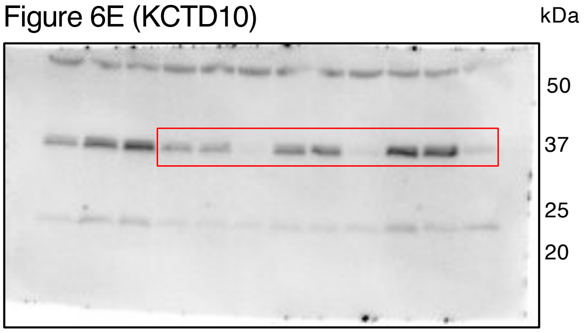

Nishiyama, Maekawa *et al*.

Full images of western blotting. The area enclosed in red squares are shown in main figures.

Figure 6F (pEGFR)

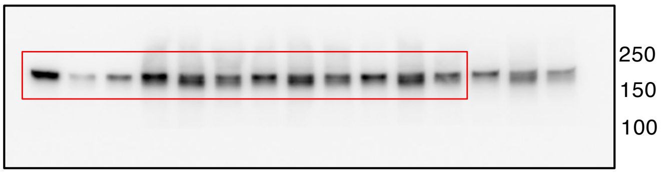

Figure 6F (pHER2)

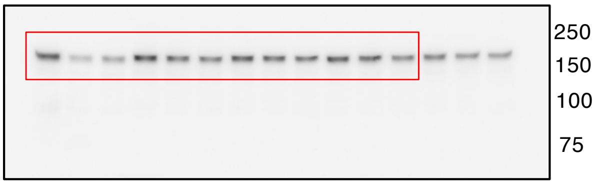

Figure 6F (EGFR)

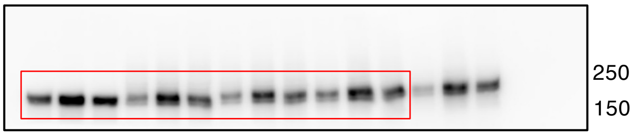

Figure 6F(HER2)

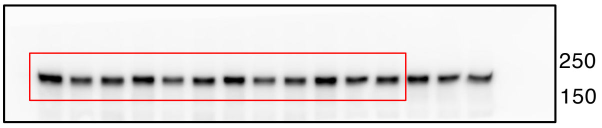

Figure 6F (pAkt)

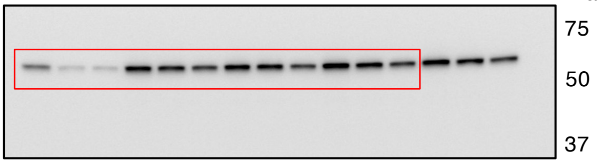

Figure 6F (pERK-1/2)

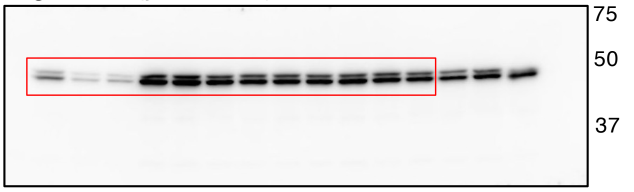

Figure 6F (Akt)

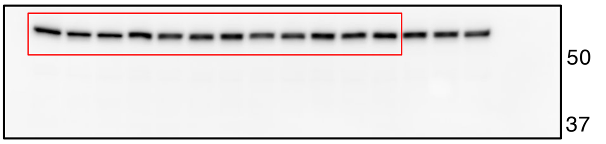

Figure 6F (ERK-1/2)

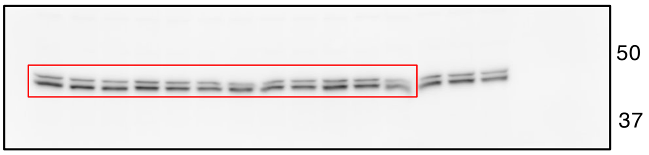

Figure 6F (CUL3)

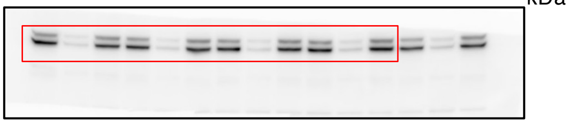

Figure 6F (KCTD10)

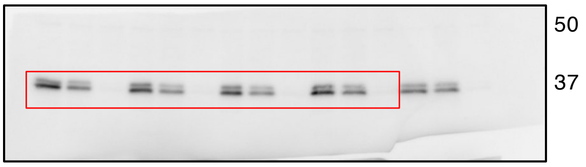

Figure 6F (RhoB)

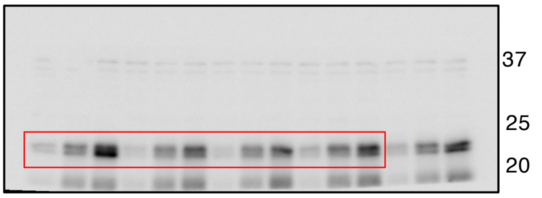

Figure 6F (GAPDH)

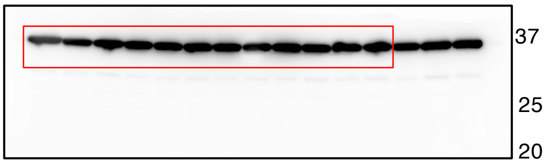

Nishiyama, Maekawa *et al*.

Full images of western blotting. The area enclosed in red squares are shown in main figures.

Figure 7B (pEGFR)

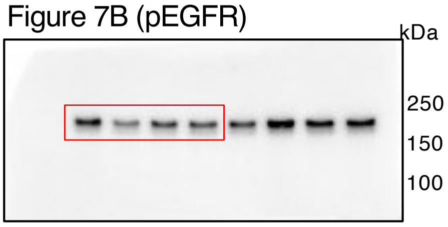

Figure 7B (EGFR)

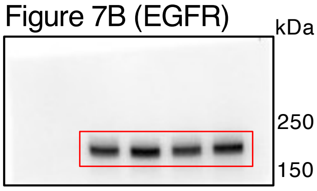

Figure 7B (KCTD10)

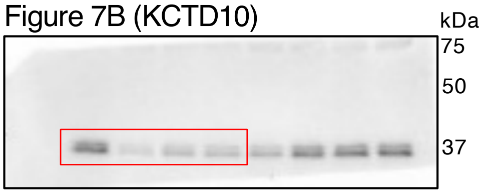

Figure 7B (pHER2)

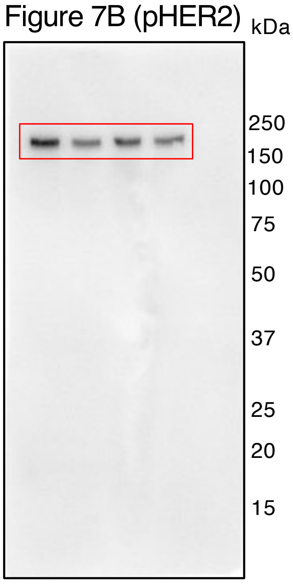

Figure 7B (HER2)

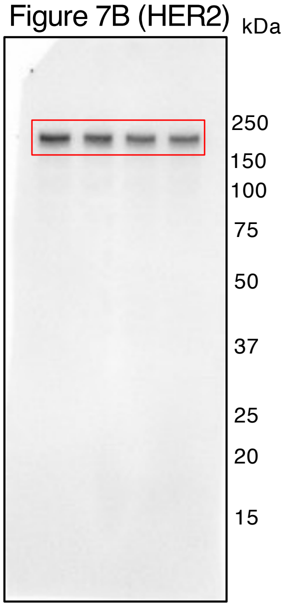

Figure 7B (RhoB)

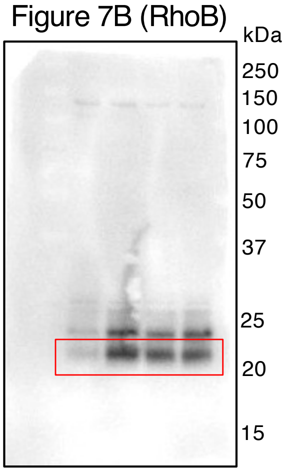

Figure 7B (GAPDH)

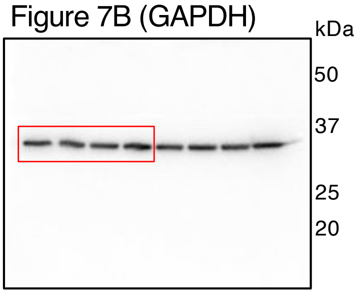

Figure 7B (CNKSR1)

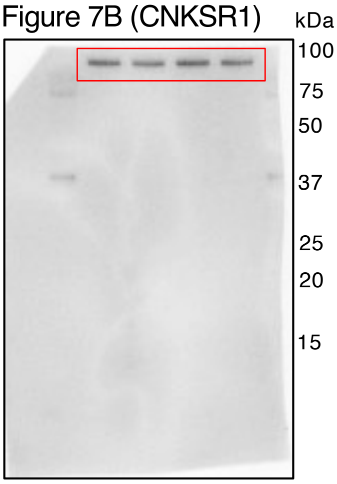

Figure 7B (PTPRH)

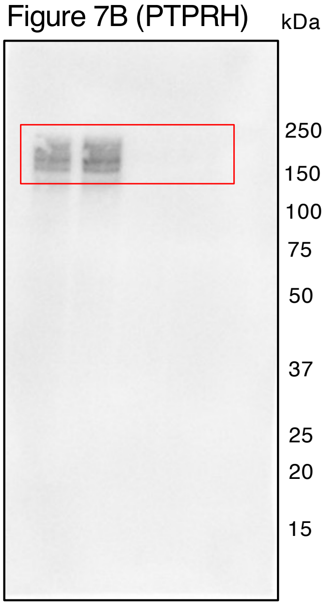

Figure 7D (FLAG)

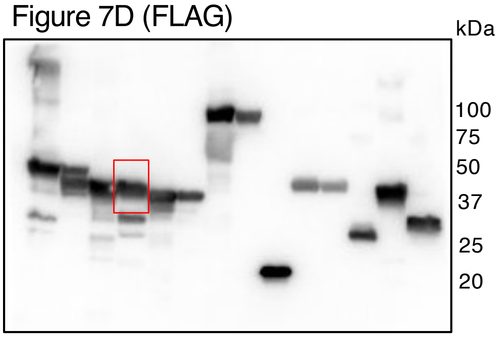

Nishiyama, Maekawa *et al.*

Full images of western blotting. The area enclosed in red squares are shown in main figures.

Figure 7F (pEGFR)

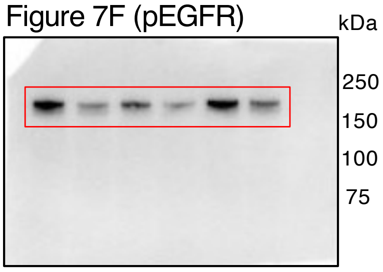

Figure 7F (EGFR)

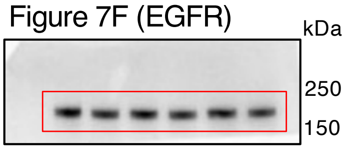

Figure 7F (pHER2)

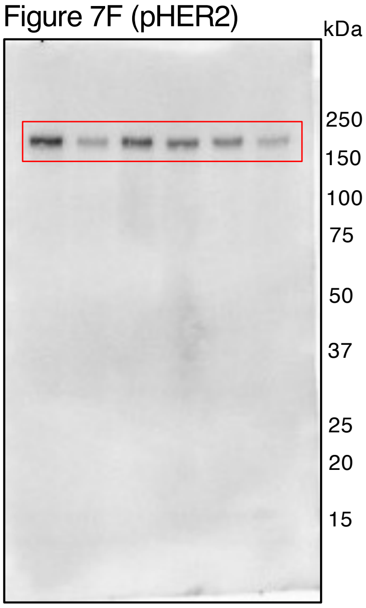

Figure 7F (HER2)

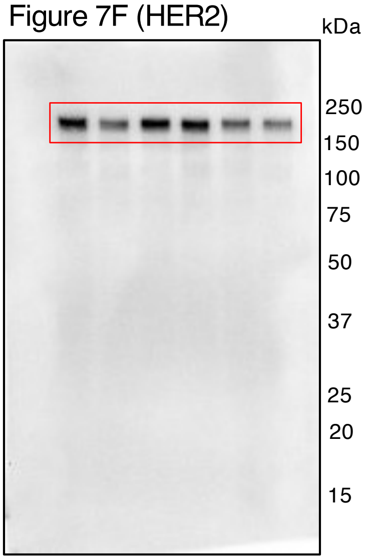

Figure 7F (CNKSR1)

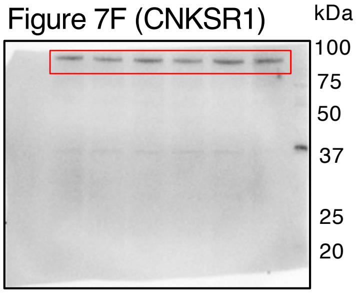

Figure 7F (KCTD10)

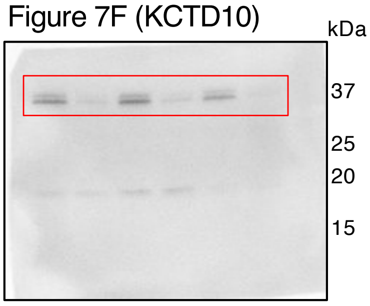

Figure 7F (HA)

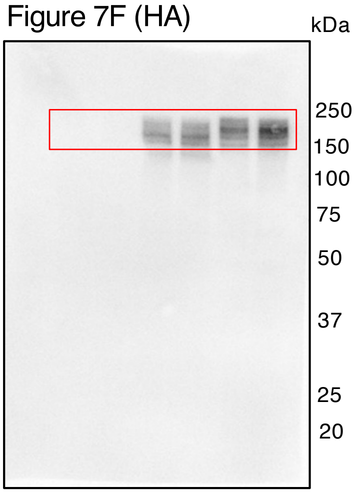

Figure 7F (RhoB)

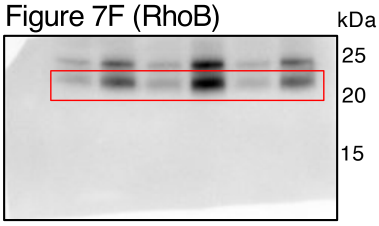

Figure 7F (GAPDH)

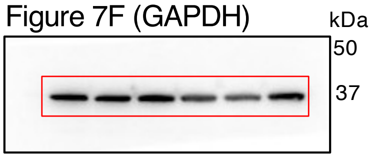

Nishiyama, Maekawa *et al.*

Full images of western blotting. The area enclosed in red squares are shown in main figures.

Figure 7I (HA)

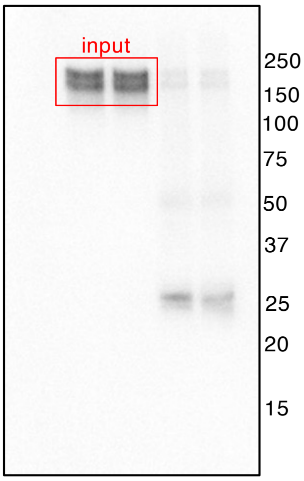

Figure 7I (CNKSR1)

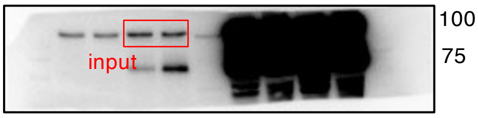

Figure 7I (CNKSR1)

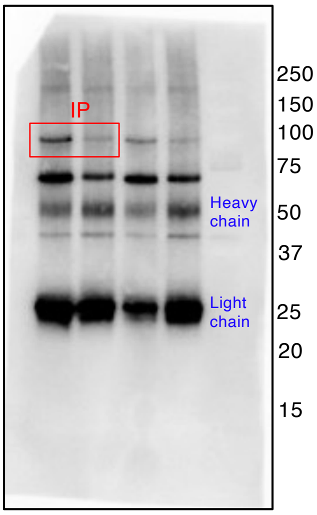

Figure 7I (HA)

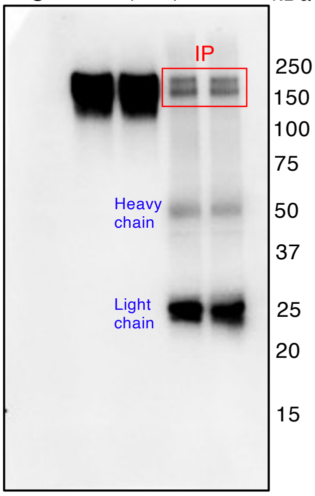

Figure 7I (RhoB)

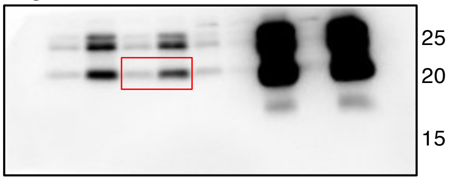

Figure 8B (FLAG)

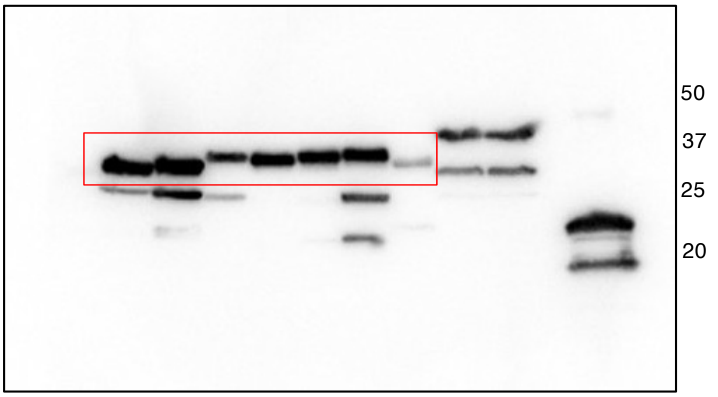

Figure 7I (KCTD10)

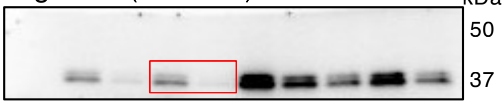

Nishiyama, Maekawa *et al*.

Full images of western blotting. The area enclosed in red squares are shown in main figures.

Figure S2 (pEGFR)

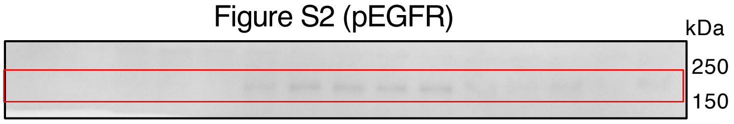

Figure S2 (pHER2)

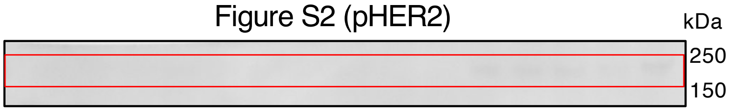

Figure S2 (RhoB)

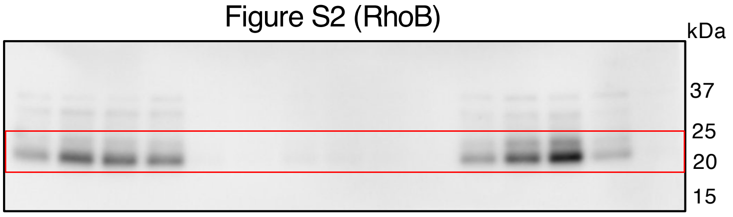

Figure S2 (KCTD10)

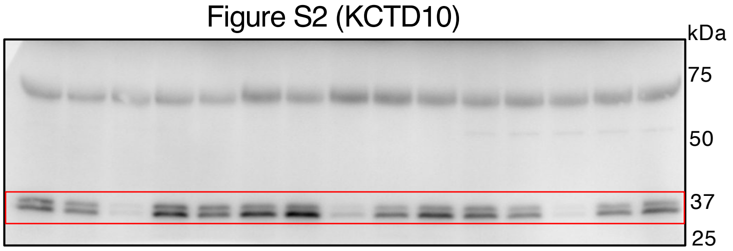

Figure S2 (GAPDH)

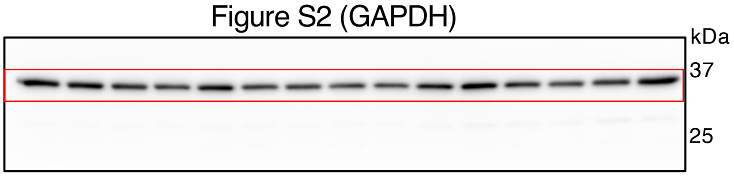

Figure S2 (EGFR)

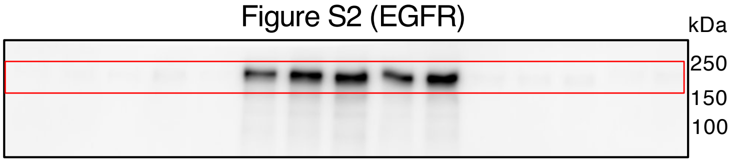

Figure S2(HER2)

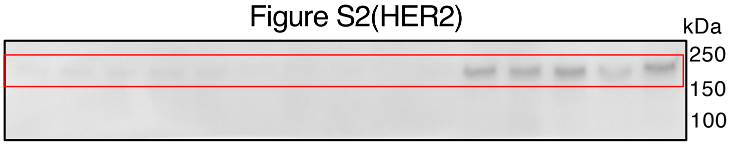

Figure S2 (CUL3)

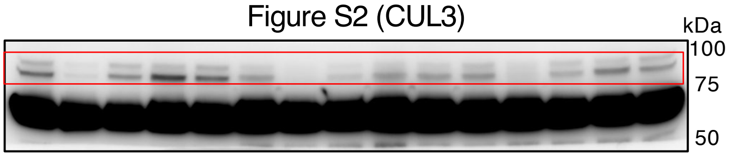

Figure S2 (CNKSR1)

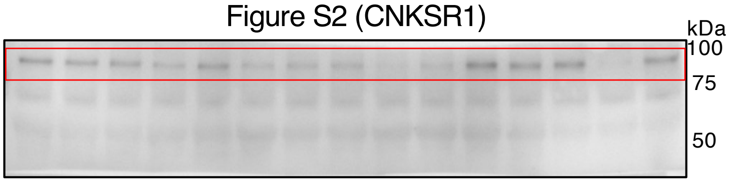

Nishiyama, Maekawa *et al*.

Full images of western blotting. The area enclosed in red squares are shown in main figures.

Figure S4 (pEGFR)

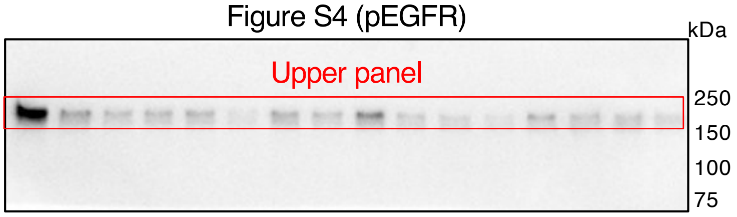

Figure S4 (pEGFR)

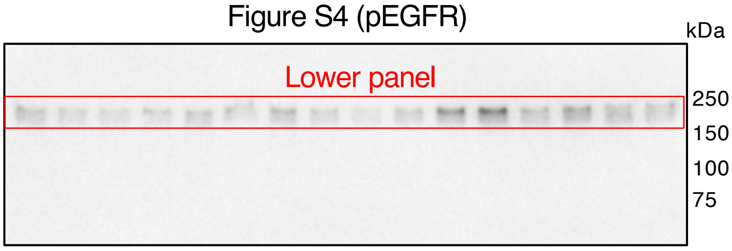

Figure S4 (EGFR)

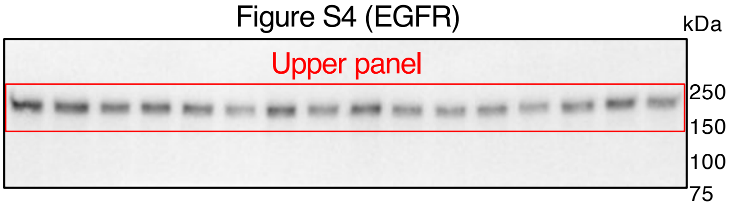

Figure S4 (EGFR)

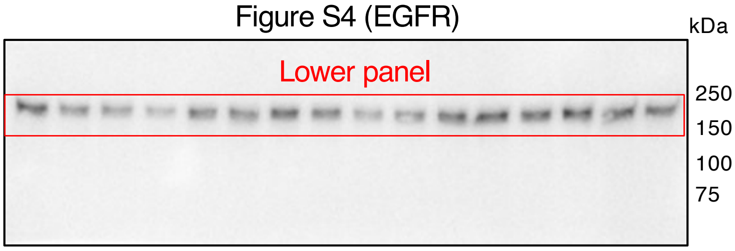

Figure S4 (KCTD10)

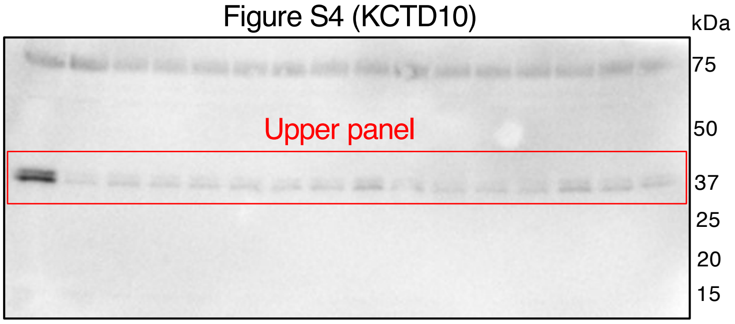

Figure S4 (KCTD10)

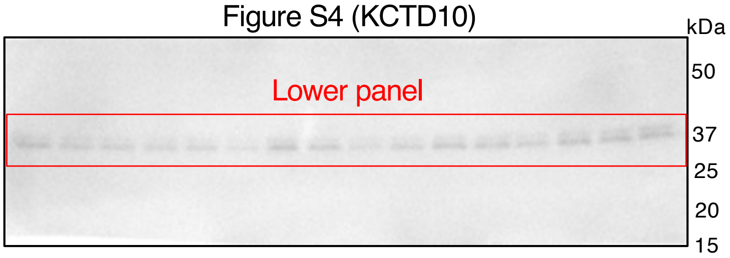

Figure S4 (GAPDH)

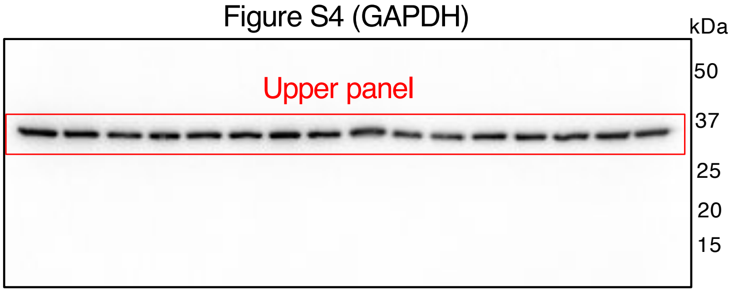

Figure S4 (GAPDH)

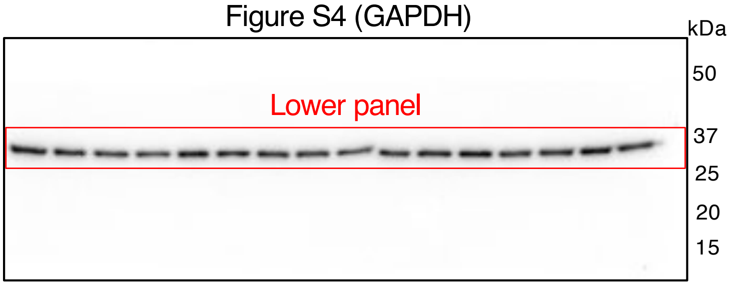

Figure S4 (pHER2)

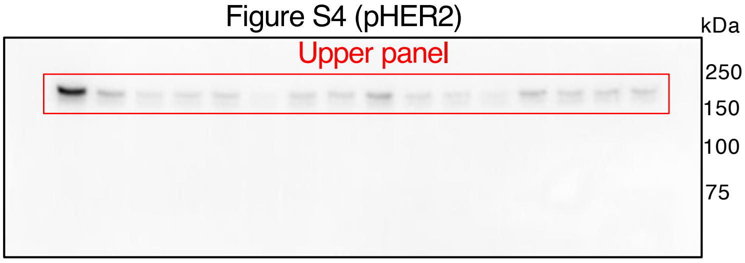

Figure S4 (pHER2)

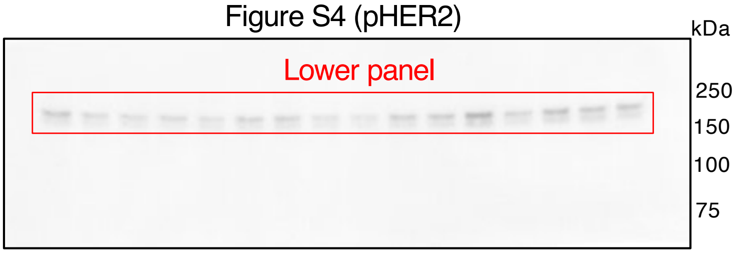

Figure S4 (HER2)

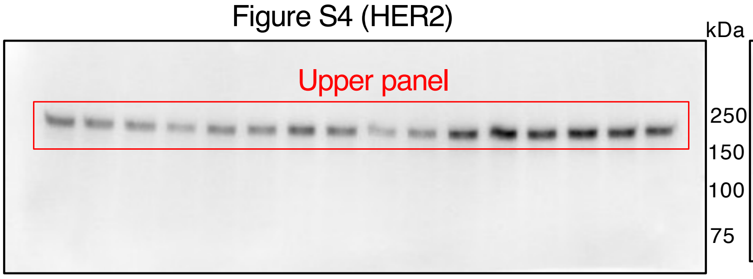

Figure S4 (HER2)

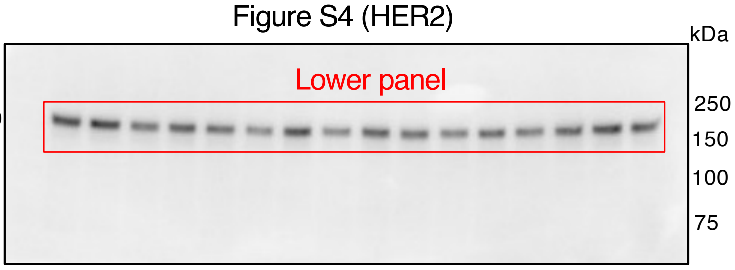

Nishiyama, Maekawa *et al*.

Full images of western blotting. The area enclosed in red squares are shown in main figures.

Figure S7A (pEGFR)

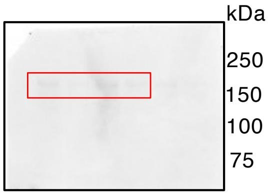

Figure S7A (EGFR)

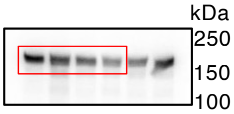

Figure S7A (KCTD10)

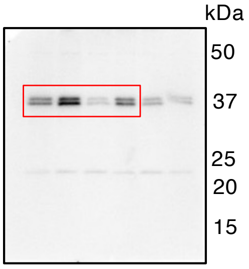

Figure S7A (CUL3)

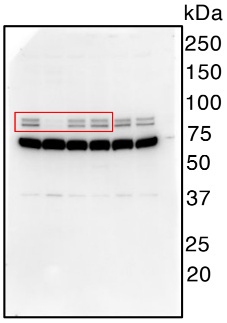

Figure S7A (RhoB)

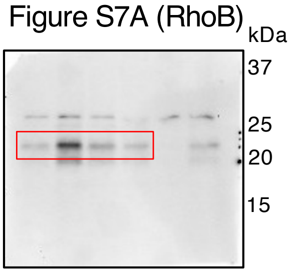

Figure S7A (CNKSR1)

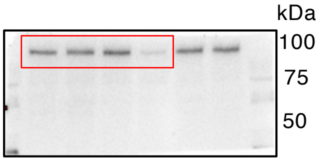

Figure S7A (PTPRH)

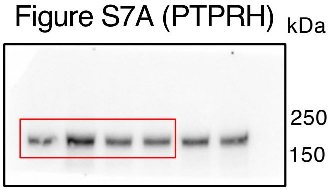

Figure S7A (GAPDH)

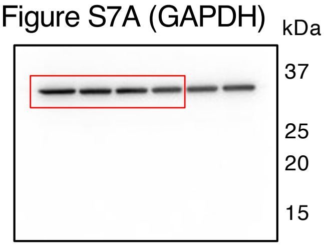

Figure S7B, C (EGFR)

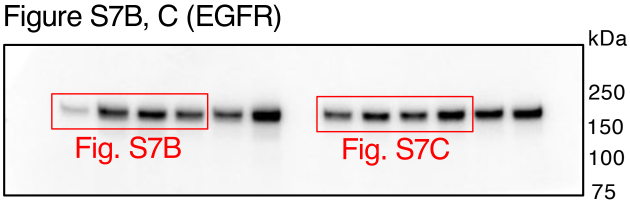

Figure S7B, C (pEGFR)

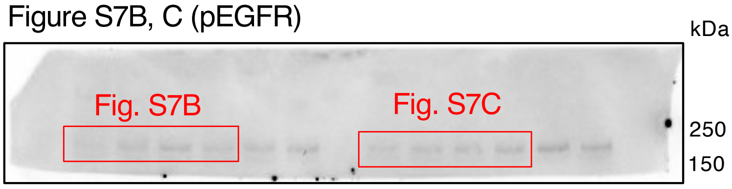

Figure S7B, C (GAPDH)

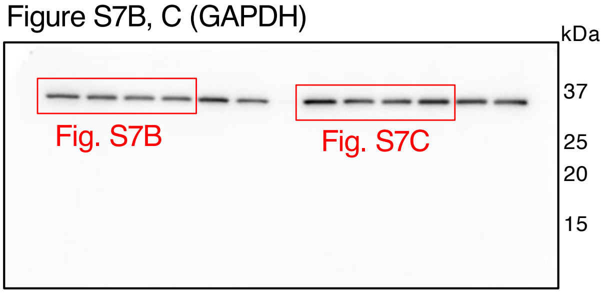

Figure S7B, C (CUL3)

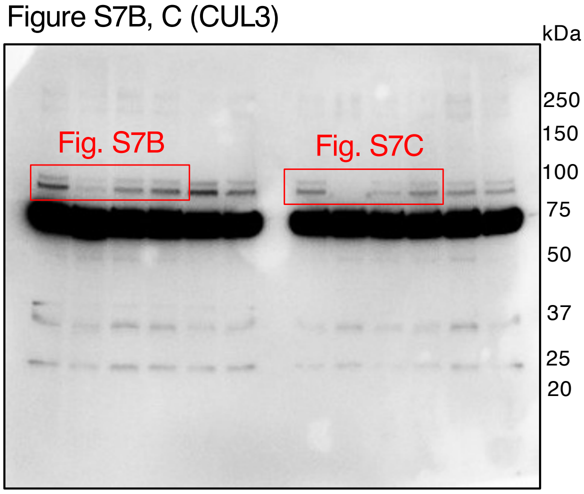

Figure S7B, C (KCTD10)

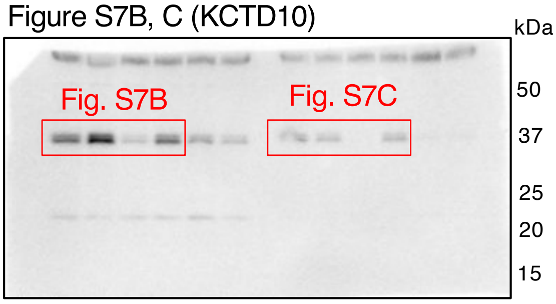

Figure S7B, C (CNKSR1)

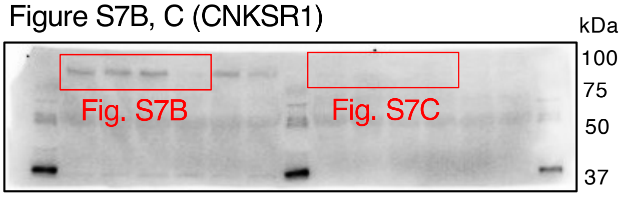

Figure S7B, C (PTPRH)

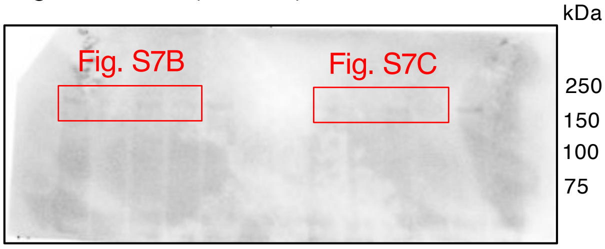

Figure S7B, C (RhoB)

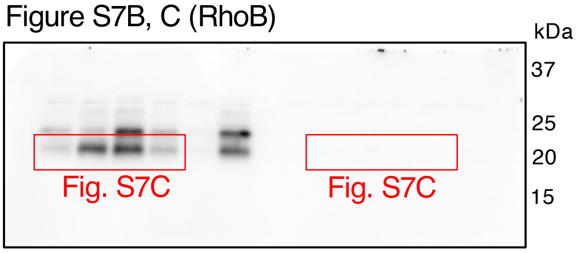

Supplement: Supplementary file 1 [file LSA-2021-01095_SdataF1_F2_F3_F5_F6_F7_F8_FS2_FS4_FS7.pdf]
